# Large Bowel Obstruction Secondary to a Fecaloma in a Child With Cerebral Palsy

**DOI:** 10.7759/cureus.31078

**Published:** 2022-11-04

**Authors:** Sabeen Ul Haq

**Affiliations:** 1 Family Medicine, King Abdulaziz Medical City, Jeddah, SAU

**Keywords:** trappc9, radiology, cerebral palsy, family medicine, pediatrics, constipation, fecaloma

## Abstract

A nine-year-old wheelchair-bound female with cerebral palsy and intellectual disability secondary to trafficking protein particle complex subunit 9 (*TRAPPC9*) mutation presented to the family medicine clinic after not having passed stool for six days. There was a history of chronic constipation. Examination revealed high-pitched “tinkling” bowel sounds; therefore, a plain abdominal X-ray was ordered to rule out the possibility of intestinal obstruction, which showed a large fecaloma in the rectum with dilated bowel loops proximal to it, signifying obstruction. This was successfully treated with the administration of a rectal enema and confirmed by a post-enema radiograph. Although rare in children, a fecaloma should be considered a cause of bowel obstruction, especially where there is a history of chronic constipation. A plain abdominal X-ray can be useful in diagnosing a fecaloma in pediatric cases.

## Introduction

Constipation is a problem that commonly affects children [1] with a prevalence of 5%-30% in the child population [2]. Constipation lasting longer than eight weeks is classed as chronic [2]. Colonic transit time is prolonged in individuals with intellectual disabilities, leading to constipation; however, the pathophysiology is poorly understood [3]. Furthermore, children with cerebral palsy are more at risk of suffering from constipation due to poor mobility [2] and food intake patterns [4]. The majority of children with constipation usually present to their general practitioner (GP) or family medicine physician [5]; therefore, it is essential for primary care doctors to be aware of the possible complications of constipation, including rare ones, such as a fecaloma, which can present with nonspecific symptoms [6]. A clear history and examination are essential and will influence further investigations and management.

## Case presentation

**Figure 1:**
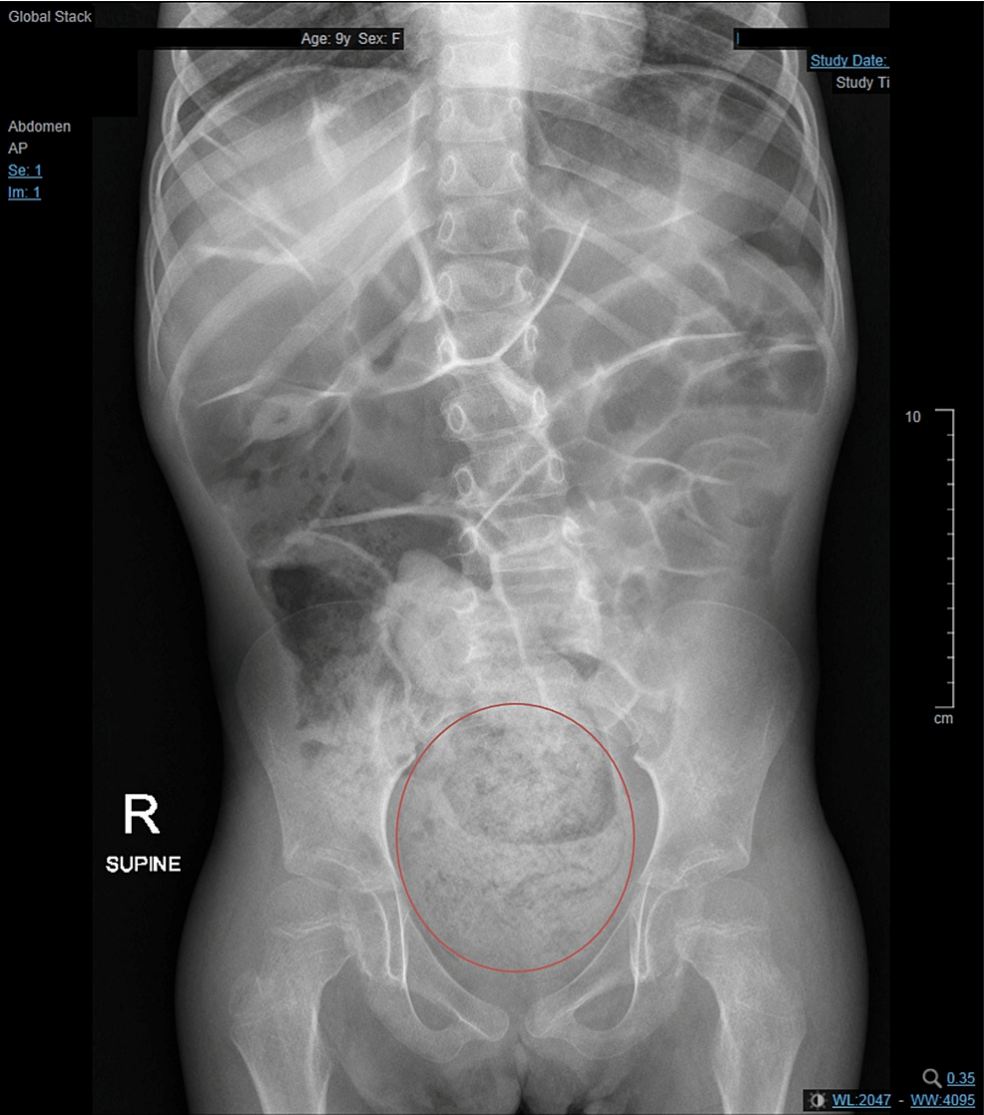
Fecaloma (circled in red) and dilated large bowel loops

**Figure 2:**
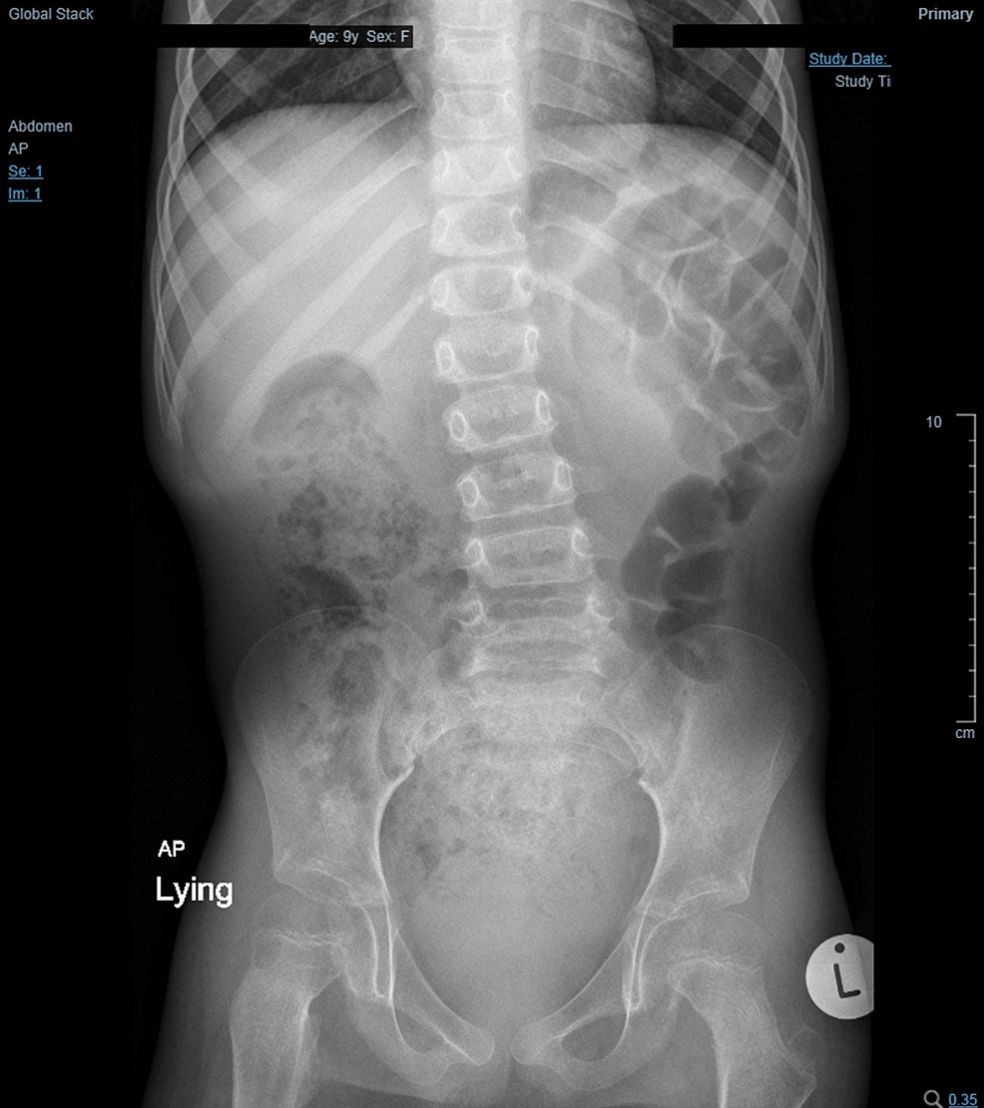
Post-enema radiograph showing the resolution of the fecaloma and dilated bowel loops

A nine-year-old female with asthma, cerebral palsy, and intellectual disability secondary to trafficking protein particle complex subunit 9 (*TRAPPC9*) mutation causing severe developmental and language delay was brought to the family medicine clinic by her mother. The presenting complaint was that the child had not passed stool for six days. She was prone to constipation and had been treated for this in the past with oral laxatives. On this occasion, she had been given a single sachet of macrogol (polyethylene glycol) but without effect. There was no history of fever, vomiting, or abdominal pain. She was eating and drinking well and passing urine. The mother could not comment on whether the child was passing flatus. There was no family history of bowel disorders or constipation. On examination in her wheelchair, she was not in any distress, and vital signs were within normal parameters for her age. Her abdomen was soft, non-tender, and non-distended, and no abdominal masses could be felt in the sitting position. Auscultation revealed high-pitched “tinkling” bowel sounds; therefore, a plain abdominal X-ray was ordered to check for intestinal obstruction, and this showed a large fecaloma in the rectum with dilated bowel loops proximal to it as a sign of a mechanical large bowel obstruction (Figure 1). The child received a rectal (pediatric fleet) enema, administered by the nurse, in the holding bay area. Shortly afterward, she passed a large amount of stool. A repeat abdominal examination was unremarkable, and a repeat abdominal X-ray showed the resolution of the fecaloma and the resulting large bowel obstruction (Figure 2). She was discharged home on a maintenance course of pediatric macrogol (polyethylene glycol).

## Discussion

Constipation is common in children [1], and the etiology can be multifactorial [2]. Children with cerebral palsy are at greater risk of suffering from constipation [2,4]. A fecaloma is a hard mass that results as a complication of fecal impaction [7]. Calcium soaps deposit in layers to form a laminated mass [8]. It is a rare complication of chronic constipation and can present with vague symptoms [6]. Obstruction, perforation, ulceration, and hydronephrosis have been reported in the literature as complications of a fecaloma [8].

A detailed history and plain X-ray are usually sufficient to aid the diagnosis of a fecaloma [7]. Evidence shows that auscultation of bowel sounds is not a very specific method of diagnosing bowel obstruction [9] and that clinical decisions in patients with suspected bowel obstruction should not be based on an auscultatory assessment of bowel sounds due to its low accuracy [10]. However, in this case, where the patient did not present with an abdominal mass or specific symptoms of bowel obstruction, the presence of “tinkling” or high-pitched bowel sounds prompted further investigation with an abdominal X-ray to check for bowel obstruction, which helped uncover the fecaloma and guide the management toward the immediate use of an enema to relieve the obstruction, instead of discharging the patient home on oral laxatives alone [11].

## Conclusions

The importance of a thorough history and examination cannot be underestimated, especially in the case of a child with a learning disability or those prone to chronic constipation. Fecalomas are rare but should be considered as a cause of constipation, especially where the history or examination points toward bowel obstruction. An abdominal X-ray is a useful investigation for the diagnosis of a fecaloma in pediatric cases. Fecalomas can be successfully treated with a combination of polyethylene glycol and sodium picosulfate, which produce fecal disimpaction in chronically constipated children. The initial management of fecalomas comprises oral laxative and enema use; however, in extreme cases, manual disimpaction, endoscopy, or surgical resection may be necessary.
